# Potassium trifluoro­[(*Z*)-3-(oxan-2-yl­oxy)prop-1-en-1-yl]borate monohydrate

**DOI:** 10.1107/S1600536808042931

**Published:** 2008-12-24

**Authors:** Julio Zukerman-Schpector, Rafael C. Guadagnin, Hélio A. Stefani, Lorenzo do Canto Visentin

**Affiliations:** a Department of Chemistry, Universidade Federal de São Carlos, 13565-905 São Carlos, SP, Brazil; b Departamento de Farmácia, Faculdade de Ciências Farmacêuticas, Universidade de São Paulo, São Paulo-SP, Brazil; c Instituto de Química, Universidade Federal do Rio de Janeiro-RJ, Brazil

## Abstract

The title compound, K^+^·C_8_H_13_BF_3_O_2_^−^·H_2_O, which was obtained from the reaction of a modified form of *Z*-vinylic telluride *via* a transmetalation reaction with *n*-BuLi, crystallizes as K^+^ and C_8_H_13_BF_3_O_2_^−^ ions along with a water mol­ecule. The K^+^ cation is surrounded by four anions, making close contacts with six F atoms at 2.659 (3)–2.906 (3) Å and with two O atoms at 2.806 (3) and 2.921 (3) Å in a distorted bicapped trigonal-prismatic geometry.

## Related literature

For related structures, see: Stefani *et al.* (2006 ▶); Caracelli *et al.* (2007 ▶); Zukerman-Schpector *et al.* (2008 ▶). For related literature, see: Vieira *et al.* (2008 ▶). For the synthesis, see: Bernady *et al.* (1979 ▶). For ring puckering analysis, see: Cremer & Pople (1975 ▶).
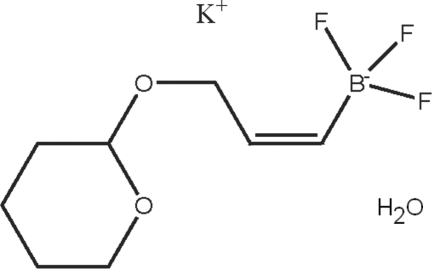


## Experimental

### 

#### Crystal data

K^+^·C_8_H_13_BF_3_O_2_^−^·H_2_O
*M* *_r_* = 266.11
Orthorhombic, 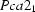

*a* = 8.5210 (7) Å
*b* = 17.056 (1) Å
*c* = 8.6318 (7) Å
*V* = 1254.50 (16) Å^3^
*Z* = 4
Mo *K*α radiation
μ = 0.45 mm^−1^
*T* = 291 (2) K
0.27 × 0.10 × 0.04 mm

#### Data collection

Nonius KappaCCD diffractometer
Absorption correction: multi-scan (*SADABS*; Bruker, 2006 ▶) *T*_min_ = 0.888, *T*_max_ = 0.982
11914 measured reflections
2324 independent reflections
1604 reflections with *I* > 2σ(*I*)
*R*_int_ = 0.074

#### Refinement

*R*[*F* ^2^ > 2σ(*F* ^2^)] = 0.041
*wR*(*F* ^2^) = 0.096
*S* = 1.03
2324 reflections
145 parameters
1 restraint
H-atom parameters constrained
Δρ_max_ = 0.23 e Å^−3^
Δρ_min_ = −0.19 e Å^−3^
Absolute structure: Flack (Flack, 1983 ▶), 1064 Friedel pairs
Flack parameter: 0.07 (9)

### 

Data collection: *COLLECT* (Nonius, 1998 ▶); cell refinement: *PHICHI* (Duisenberg *et al.*, 2000 ▶); data reduction: *EVAL-14 (CCD)* (Duisenberg *et al.*, 2003 ▶); program(s) used to solve structure: *SIR97* (Altomare *et al.*, 1999 ▶); program(s) used to refine structure: *SHELXL97* (Sheldrick, 2008 ▶); molecular graphics: *ORTEP-3 for Windows* (Farrugia, 1997 ▶); software used to prepare material for publication: *WinGX* (Farrugia, 1999 ▶).

## Supplementary Material

Crystal structure: contains datablocks global, I. DOI: 10.1107/S1600536808042931/ng2525sup1.cif

Structure factors: contains datablocks I. DOI: 10.1107/S1600536808042931/ng2525Isup2.hkl

Additional supplementary materials:  crystallographic information; 3D view; checkCIF report

## Figures and Tables

**Fig. 1.**
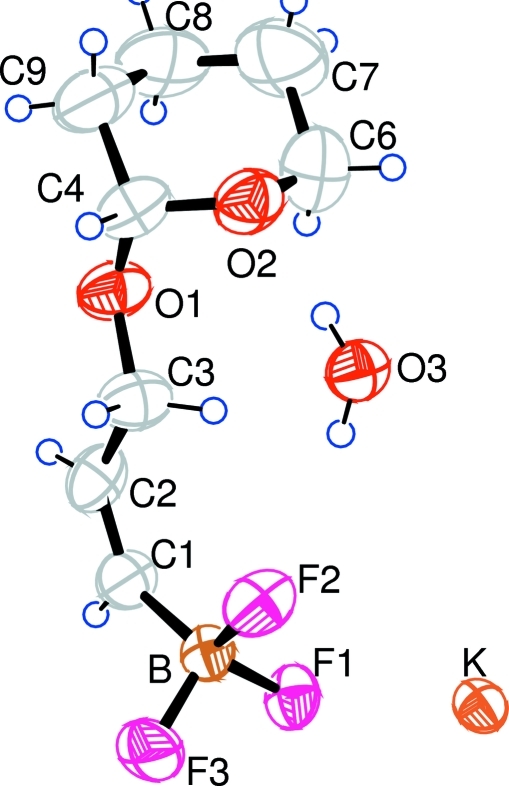
The molecular structure of the title compound showing atom labelling scheme and displacement ellipsoids at the 50% probability level (arbitrary spheres for the H atoms).

**Fig. 2.**
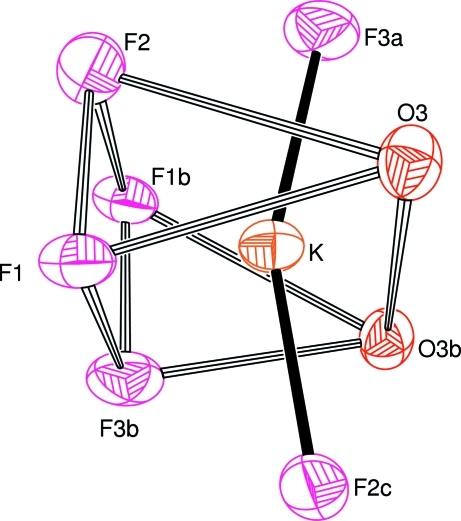
The bicapped trigonal prism around the K+ ion. Symmetry operations: a = 1 - x, -y, z - 1/2; b = 1/2 - x, y, z - 1/2; c = x - 1/2, -y, z.

## supplementary crystallographic information

### Comment

Organic compounds of tellurium, such as *Z*-vinylic tellurides, are important synthetic precursors of organometallic molecules and organic salts and can be useful in the synthesis of new potassium vinyl trifluoroborate salts. Organotrifluoroborates represent an alternative to boronic acids, boronate esters, and organoboranes for use in the Suzuki-Miyaura reaction and other transition-metal-catalyzed cross-coupling reactions (Vieira *et al.* 2008). The title compound (I), Fig. 1, was studied as part of an ongoing systematic synthesis of trifluoroborate compounds (Stefani *et al.*(2006), Caracelli *et al.* (2007); Zukerman-Schpector *et al.* (2008)). The oxane ring is in a slightly distorted chair conformation, the ring-puckering parameters (Cremer & Pople, 1975) are q_2_ = 0.033 (6) Å, q_3_ = 0.555 (6) Å, Q = 0.556 (7)°, θ = 3.4 (6)° and φ_2_ = 156 (11)°. The geometry around the K^+^ ion can be described as a distorted bicaped trigonal prism as shown in Figure 2. Besides the K^+^ interactions,the molecules are connected *via* C3···O2^i^ = 3.600 (6) Å, C3—H3A···O2^i^ = 132° (i = -*x* + 3/2, *y*, *z* + 1/2) contact.

### Experimental

The starting propargylic alcohol was protected with dihydropyran (Bernady *et al.* 1979) and *via* hydrotelluration of the alkyne transformed in the correspondent *Z*-vinylic telluride. Next, nBuLi (0.8 mmol) was added dropwise at 203 K to a solution of the *Z*-vinylic telluride (1 mmol) in Et_2_O (6 ml). The bath temperature was raised to 253 K. After 20 minutes B(OiPr)_3_ (1.0 mmol) was added at 233 K. After 1 h, an aqueous solution of KHF_2_ (4 mmol in 10 ml of water) was added to the reaction mixture. Then, the solvent and water were eliminated by evaporation. To the obtained solid hot acetone was added and the bulk reactional was filtered and dried, yielding 24% of (*Z*)-potassium vinyltrifluoroborate salt. Single crystals were obtained by slow evaporation from Et_2_O.

### Refinement

The H atoms were refined in the riding-model approximation with *U*_iso_(H) = 1.2*U*_eq_, and with C—H = 0.93 - 0.97 Å. The water molecule H atoms were refined riding in the position found in a difference map.

### Crystal data

**Table:**

K^+^·C_8_H_13_BF_3_O_2_^−^·H_2_O	*F*(000) = 552
*M**_r_* = 266.11	*D*_x_ = 1.409 Mg m^−^^3^
Orthorhombic, *P**c**a*2_1_	Mo *K*α radiation, λ = 0.71073 Å
Hall symbol: P 2c -2ac	Cell parameters from 9536 reflections
*a* = 8.5210 (7) Å	θ = 2.3–21.8°
*b* = 17.056 (1) Å	µ = 0.45 mm^−^^1^
*c* = 8.6318 (7) Å	*T* = 291 K
*V* = 1254.50 (16) Å^3^	Plate, colourless
*Z* = 4	0.27 × 0.10 × 0.04 mm

### Data collection

**Table:**

Nonius KappaCCD diffractometer	2324 independent reflections
Radiation source: fine-focus sealed tube	1604 reflections with *I* > 2σ(*I*)
graphite	*R*_int_ = 0.074
φ and ω scans	θ_max_ = 25.5°, θ_min_ = 3.4°
Absorption correction: multi-scan (*SADABS*; Bruker, 2006)	*h* = −10→10
*T*_min_ = 0.888, *T*_max_ = 0.982	*k* = −20→20
11914 measured reflections	*l* = −10→10

### Refinement

**Table:**

Refinement on *F*^2^	Secondary atom site location: difference Fourier map
Least-squares matrix: full	Hydrogen site location: inferred from neighbouring sites
*R*[*F*^2^ > 2σ(*F*^2^)] = 0.041	H-atom parameters constrained
*wR*(*F*^2^) = 0.096	*w* = 1/[σ^2^(*F*_o_^2^) + (0.0438*P*)^2^] where *P* = (*F*_o_^2^ + 2*F*_c_^2^)/3
*S* = 1.03	(Δ/σ)_max_ < 0.001
2324 reflections	Δρ_max_ = 0.23 e Å^−^^3^
145 parameters	Δρ_min_ = −0.19 e Å^−^^3^
1 restraint	Absolute structure: Flack (Flack, 1983), 1064 Friedel pairs
Primary atom site location: structure-invariant direct methods	Flack parameter: 0.07 (9)

### Special details

**Table:**

Geometry. All e.s.d.'s (except the e.s.d. in the dihedral angle between two l.s. planes) are estimated using the full covariance matrix. The cell e.s.d.'s are taken into account individually in the estimation of e.s.d.'s in distances, angles and torsion angles; correlations between e.s.d.'s in cell parameters are only used when they are defined by crystal symmetry. An approximate (isotropic) treatment of cell e.s.d.'s is used for estimating e.s.d.'s involving l.s. planes.

### Fractional atomic coordinates and isotropic or equivalent isotropic displacement parameters (Å^2^)

**Table:**

	*x*	*y*	*z*	*U*_iso_*/*U*_eq_	
K	0.24966 (12)	−0.03852 (4)	0.49817 (17)	0.04270 (18)	
B	0.3952 (4)	0.1021 (2)	0.7488 (8)	0.0421 (8)	
F1	0.2390 (2)	0.07214 (10)	0.7452 (5)	0.0524 (4)	
F2	0.4623 (4)	0.08281 (14)	0.6069 (3)	0.0627 (8)	
F3	0.4710 (3)	0.05485 (16)	0.8649 (3)	0.0714 (9)	
O1	0.5509 (3)	0.32819 (14)	0.5091 (4)	0.0665 (8)	
O2	0.5808 (3)	0.28932 (14)	0.2496 (5)	0.0675 (7)	
O3	0.3979 (3)	−0.12040 (12)	0.7392 (4)	0.0516 (6)	
H1O3	0.4374	−0.1060	0.8252	0.062*	
H2O3	0.4006	−0.1711	0.7375	0.062*	
C1	0.4036 (5)	0.1914 (2)	0.7978 (4)	0.0585 (12)	
H1	0.3581	0.2026	0.8932	0.070*	
C2	0.4642 (5)	0.2527 (2)	0.7267 (6)	0.0614 (11)	
H2	0.4563	0.3010	0.7758	0.074*	
C3	0.5447 (6)	0.2502 (3)	0.5726 (5)	0.0673 (12)	
H3A	0.6502	0.2297	0.5846	0.081*	
H3B	0.4877	0.2159	0.5028	0.081*	
C4	0.6461 (6)	0.3325 (2)	0.3741 (6)	0.0677 (12)	
H4	0.7499	0.3110	0.3982	0.081*	
C6	0.4336 (7)	0.3192 (3)	0.1981 (7)	0.101 (2)	
H6A	0.3585	0.3169	0.2824	0.121*	
H6B	0.3944	0.2868	0.1143	0.121*	
C7	0.4488 (8)	0.4031 (4)	0.1427 (8)	0.114 (2)	
H7A	0.3468	0.4229	0.1115	0.137*	
H7B	0.5183	0.4054	0.0538	0.137*	
C8	0.5146 (8)	0.4530 (3)	0.2740 (10)	0.100 (2)	
H8A	0.5340	0.5059	0.2372	0.120*	
H8B	0.4392	0.4558	0.3580	0.120*	
C9	0.6638 (8)	0.4176 (3)	0.3308 (7)	0.0904 (17)	
H9A	0.6999	0.4467	0.4205	0.108*	
H9B	0.7431	0.4223	0.2507	0.108*	

### Atomic displacement parameters (Å^2^)

**Table:**

	*U*^11^	*U*^22^	*U*^33^	*U*^12^	*U*^13^	*U*^23^
K	0.0363 (3)	0.0543 (4)	0.0374 (3)	−0.0017 (5)	0.0004 (3)	0.0036 (7)
B	0.0300 (17)	0.053 (2)	0.0437 (18)	−0.0018 (16)	0.007 (3)	−0.001 (3)
F1	0.0330 (9)	0.0674 (10)	0.0569 (10)	−0.0045 (9)	0.0018 (15)	−0.005 (2)
F2	0.0665 (17)	0.0580 (15)	0.0635 (15)	−0.0067 (13)	0.0313 (13)	−0.0131 (13)
F3	0.0493 (17)	0.0804 (18)	0.085 (2)	−0.0009 (15)	−0.0228 (15)	0.0288 (16)
O1	0.080 (2)	0.0450 (14)	0.0746 (19)	0.0016 (14)	0.010 (2)	0.0035 (17)
O2	0.0800 (18)	0.0491 (14)	0.0735 (16)	−0.0009 (14)	0.004 (2)	−0.002 (2)
O3	0.0605 (14)	0.0446 (13)	0.0497 (13)	0.0003 (11)	0.0093 (17)	−0.0057 (18)
C1	0.073 (3)	0.056 (3)	0.046 (2)	−0.007 (2)	0.0120 (19)	−0.0077 (17)
C2	0.066 (2)	0.049 (2)	0.069 (3)	−0.0020 (19)	0.008 (3)	−0.019 (2)
C3	0.079 (3)	0.053 (3)	0.070 (3)	0.003 (2)	0.015 (2)	0.003 (2)
C4	0.060 (3)	0.057 (3)	0.086 (3)	−0.008 (2)	0.014 (2)	0.005 (3)
C6	0.093 (4)	0.108 (4)	0.101 (5)	−0.014 (3)	−0.016 (3)	−0.008 (3)
C7	0.115 (6)	0.105 (5)	0.123 (6)	0.024 (4)	−0.017 (4)	0.034 (5)
C8	0.133 (5)	0.053 (3)	0.113 (6)	0.023 (3)	0.015 (4)	0.012 (3)
C9	0.106 (5)	0.067 (3)	0.098 (4)	−0.022 (3)	0.021 (3)	0.004 (3)

### Geometric parameters (Å, °)

**Table:**

B—F1	1.426 (4)	C3—H3B	0.9700
B—F2	1.392 (6)	C4—C9	1.507 (6)
B—F3	1.439 (6)	C4—H4	0.9800
B—C1	1.582 (5)	C6—C7	1.514 (8)
O1—C4	1.422 (5)	C6—H6A	0.9700
O1—C3	1.439 (5)	C6—H6B	0.9700
O2—C4	1.417 (6)	C7—C8	1.525 (10)
O2—C6	1.425 (6)	C7—H7A	0.9700
O3—H1O3	0.8518	C7—H7B	0.9700
O3—H2O3	0.8651	C8—C9	1.490 (8)
C1—C2	1.317 (6)	C8—H8A	0.9700
C1—H1	0.9300	C8—H8B	0.9700
C2—C3	1.497 (7)	C9—H9A	0.9700
C2—H2	0.9300	C9—H9B	0.9700
C3—H3A	0.9700		
			
F2—B—F1	106.2 (4)	C9—C4—H4	108.8
F2—B—F3	107.2 (3)	O2—C6—C7	111.2 (4)
F1—B—F3	103.5 (3)	O2—C6—H6A	109.4
F2—B—C1	116.4 (3)	C7—C6—H6A	109.4
F1—B—C1	113.2 (3)	O2—C6—H6B	109.4
F3—B—C1	109.4 (4)	C7—C6—H6B	109.4
C4—O1—C3	112.3 (3)	H6A—C6—H6B	108.0
C4—O2—C6	113.4 (4)	C6—C7—C8	108.9 (5)
H1O3—O3—H2O3	107.0	C6—C7—H7A	109.9
C2—C1—B	131.1 (4)	C8—C7—H7A	109.9
C2—C1—H1	114.5	C6—C7—H7B	109.9
B—C1—H1	114.5	C8—C7—H7B	109.9
C1—C2—C3	124.8 (4)	H7A—C7—H7B	108.3
C1—C2—H2	117.6	C9—C8—C7	109.4 (5)
C3—C2—H2	117.6	C9—C8—H8A	109.8
O1—C3—C2	109.2 (3)	C7—C8—H8A	109.8
O1—C3—H3A	109.8	C9—C8—H8B	109.8
C2—C3—H3A	109.8	C7—C8—H8B	109.8
O1—C3—H3B	109.8	H8A—C8—H8B	108.2
C2—C3—H3B	109.8	C8—C9—C4	112.7 (5)
H3A—C3—H3B	108.3	C8—C9—H9A	109.0
O2—C4—O1	111.8 (3)	C4—C9—H9A	109.0
O2—C4—C9	110.6 (4)	C8—C9—H9B	109.0
O1—C4—C9	108.1 (4)	C4—C9—H9B	109.0
O2—C4—H4	108.8	H9A—C9—H9B	107.8
O1—C4—H4	108.8		
			
F2—B—C1—C2	0.1 (7)	C3—O1—C4—O2	66.2 (5)
F1—B—C1—C2	−123.4 (6)	C3—O1—C4—C9	−171.9 (4)
F3—B—C1—C2	121.8 (5)	C4—O2—C6—C7	60.0 (6)
B—C1—C2—C3	−0.3 (8)	O2—C6—C7—C8	−57.6 (7)
C4—O1—C3—C2	171.2 (4)	C6—C7—C8—C9	54.2 (7)
C1—C2—C3—O1	161.9 (4)	C7—C8—C9—C4	−53.4 (7)
C6—O2—C4—O1	63.8 (5)	O2—C4—C9—C8	53.8 (6)
C6—O2—C4—C9	−56.6 (5)	O1—C4—C9—C8	−68.9 (6)

## References

[bb1] Altomare, A., Burla, M. C., Camalli, M., Cascarano, G. L., Giacovazzo, C., Guagliardi, A., Moliterni, A. G. G., Polidori, G. & Spagna, R. (1999). *J. Appl. Cryst.* **32**, 115–119.

[bb2] Bernady, K. F., Floyd, M. B., Poletto, J. F. & Weiss, M. J. (1979). *J. Org. Chem.* **44**, 1438–1447.

[bb3] Bruker (2006). *SADABS* Bruker AXS Inc., Madison, Wisconsin, USA.

[bb4] Caracelli, I., Stefani, H. A., Vieira, A. S., Machado, M. M. P. & Zukerman-Schpector, J. (2007). *Z. Krist. New Cryst. Struct.* **222**, 345–346.

[bb5] Cremer, D. & Pople, J. A. (1975). *J. Am. Chem. Soc.* **97**, 1354–1358.

[bb6] Duisenberg, A. J. M., Hooft, R. W. W., Schreurs, A. M. M. & Kroon, J. (2000). *J. Appl. Cryst.* **33**, 893–898.

[bb7] Duisenberg, A. J. M., Kroon-Batenburg, L. M. J. & Schreurs, A. M. M. (2003). *J. Appl. Cryst.* **36**, 220–229.

[bb8] Farrugia, L. J. (1997). *J. Appl. Cryst.* **30**, 565.

[bb9] Farrugia, L. J. (1999). *J. Appl. Cryst.* **32**, 837–838.

[bb10] Flack, H. D. (1983). *Acta Cryst.* A**39**, 876–881.

[bb11] Nonius (1998) *COLLECT* Nonius BV, Delft, The Netherlands.

[bb12] Sheldrick, G. M. (2008). *Acta Cryst.* A**64**, 112–122. 10.1107/S0108767307043930 18156677

[bb13] Stefani, H. A., Cella, R., Zukerman-Schpector, J. & Caracelli, I. (2006). *Z. Krist. New Cryst. Struct.* **221**, 167–168.

[bb14] Vieira, A. S., Fiorante, P. F., Zukerman-Schpector, J., Alves, D., Botteselle, G. V. & Stefani, H. A. (2008). *Tetrahedron*, **64**, 7234–7241.

[bb15] Zukerman-Schpector, J., Guadagnin, R. C., Stefani, H. A. & Visentin, L. do C. (2008). *Acta Cryst.* E**64**, m1525. 10.1107/S1600536808036428 PMC2959975 21581142

